# Behavioral Responses of the Bumblebee *Bombus terrestris* to Volatile Compounds from Blueberries

**DOI:** 10.3390/biology14111570

**Published:** 2025-11-09

**Authors:** Yun He, Jiaru Zhang, Ziyang Hu, Yingxue Cao, Kevin H. Mayo, Duo Liu, Mingju E

**Affiliations:** 1 School of Life Sciences, Changchun Normal University, 677 Changjibei Road, Changchun 130032, China; qx202310015@stu.ccsfu.edu.cn (Y.H.);; 2 Department of Biochemistry, Molecular Biology & Biophysics, University of Minnesota Health Sciences Center, 6-155 Jackson Hall, Minneapolis, MN 55455, USA

**Keywords:** *Bombus terrestris*, *Vaccinium* spp., volatile compounds, EAG

## Abstract

Bumblebees are important pollinating insects in crop pollination. Chemical attractants can effectively improve the flower-visiting efficiency of bumblebees, thereby increasing blueberry yields. To identify volatile compounds that attract bumblebees, we collected volatile compounds from blueberry flowers using headspace extraction. Gas chromatography– mass spectrometry (GC–MS) identified 32 volatile compounds, with Linalool and Styrene being the primary substances that accounted for 25.93% and 14.28%, respectively. The olfactory threshold of bumblebee antennae was assessed using electroantennography (EAG), and the behavioral responses from bumblebees were investigated using a Y-tube olfactometer. Results indicate that among the six classes of volatiles—alcohols, aldehydes, esters, ketones, aromatic compounds, and olefins—alcohols constituted the predominant proportion. Among these, six compounds—benzaldehyde, phenylpropylaldehyde, citral, linalool, α-terpineol, and geraniol—induced significant antennal responses in bumblebees. Our assays showed that geraniol, linalool, and α-terpineol at concentrations of 0.1 μg/μL, 1 μg/μL, and 10 μg/μL elicited attraction, whereas higher concentrations of benzaldehyde, benzenepropanal, and citral had repellent effects.

## Simple Summary

Blueberries rely on insects to carry pollen between flowers, but natural pollination is often too low to ensure high fruit yields. This study explored which floral scents attract buff-tailed bumblebees, the most effective pollinators of this crop. We collected air samples from around blueberry blossoms and found 32 different fragrance compounds, with two scents, linalool and styrene, being the most common. We then tested how the bees reacted to these scents by measuring their antennal signals and observing their choices in a simple maze. The bees were drawn to low levels of certain sweet, plant-based alcohols, while high amounts of some sharp-smelling chemicals turned them away. These results identify the key scents that guide bees to flowers and open the door to creating natural sprays that can safely draw more pollinators to blueberry fields. Such sprays could help farmers produce larger harvests and higher-quality fruit without relying on harmful chemicals, supporting both agriculture and the environment.

## 1. Introduction

Bumblebees (*Bombus terrestris*), key pollinators, are large, cold-tolerant and densely furred, with excellent low-light adaptability, rapid flower-visiting speed and vigorous foraging capacity [1,2]. Their ability to perform buzz-pollination—sonication of poricidal anthers—makes them especially effective for crops such as tomato and blueberry, where pollen release otherwise limits fruit production. Bumblebees are now the standard managed pollinator for greenhouse tomatoes, peppers, strawberries, pumpkins and blueberries [3,4], and their deployment has reduced labor costs of hand pollination while increasing yield stability across multiple continents.

In the pollinator environment, most olfactory signals are volatile compounds—plant volatiles, sex pheromones, alarm pheromones, etc. [5,6,7]—synthesized by all plant organs [8] and strongly influencing insect behavior [9]. Floral volatiles not only attract pollinators but also shape their nectar-collecting choices; for example, specific aromatics increase bees’ preference for high-sugar flowers [10]. For bumblebees, aromatic or relatively strong odors are particularly attractive: E-β-bergamotene emitted by *Oxalis corniculata* L. [11] and phenylacetaldehyde from rapeseed (*Brassica rapa* L.) [12] significantly enhance visitation. Yet which volatiles mediate close-range attraction of bumblebees to blueberry flowers remains unclear.

Blueberry (*Vaccinium* spp.) is prized for its delicate flesh, antioxidant content and premium price [13,14,15], but self-pollination stands at <30% of flowers and open-field fruit pollination depends almost entirely on insects [16,17]. Field studies show that a single visit by a buzzing *B. terrestris* increases fertilization success by >30%, berry weight by 8–12%, seed number by 20–40% and the share of export-grade fruit by 15–20%, translating into increased fruit gains of 20–35% and simultaneous reductions in malformation and firmness loss [18,19]. Consequently, bumblebee visitation is the primary determinant for both volume and commercial quality for blueberry harvests.

This study investigated blueberry floral volatiles using solid-phase microextraction (SPME), identified the components by GC–MS and screened electrophysiologically active compounds using GC–EAG on bumblebee antennae. Behavioral assays were then used to quantify bee preferences for individual volatiles, with the aim of identifying the chemical basis of the blueberry–Bombus interaction and developing natural pollinator attractants to improve pollination rates, yield and berry quality.

## 2. Materials and Methods

### 2.1. Insects

Healthy female bumblebees used here were obtained from the Jilin City Bee Research Institute in Jilin Province and kept in insect rearing cages (18 cm × 18 cm × 10 cm). The lab temperature was set at 26 ± 1 °C in a red-light environment with relative humidity maintained at 60 ± 10%. Bumblebees were fed a solution of pollen and sucrose (concentration ratio 1:1.2).

### 2.2. Collection and Analysis of Headspace Volatiles from Vaccinium *spp.*

In early May 2024, during peak blueberry flowering season, we acquired samples from three blueberry plantations across Changbai Mountain at the outdoor base in Guanghua Town, Tonghua County, Baishan City, Jilin Province. From these samples, we obtained a comprehensive volatile compound profile. Nine plants (three per site) with uniform vegetative growth were selected, and 8–12 freshly opened flowers were picked from each plant. Ambient conditions at sampling averaged 65 ± 5% RH and 18 ± 2 °C, typical for early May in the Changbai mountain range. One week after anthesis, intact flowers were cut with tweezers, immediately placed in 20 mL headspace vials, and subjected to SPME (CAR/DVB/PDMS fiber) extraction at 70 °C for 30 min; the fiber was then desorbed in the GC–MS injector at 250 °C for 5 min. Each set of flowers from a single plant constituted one replicate, giving a total of nine replicates (three plants × three sites).

### 2.3. Gas Chromatography–Mass Spectrometry (GC–MS)

Extracts were analyzed by GC–MS using an HP-5MS flexible quartz capillary column (30 m × 0.25 mm × 0.25 μm Agilent Technologies, Santa Clara, CA, USA) equipped with an Agilent 7000C. The carrier gas was high-purity helium (purity ≥ 99.99%) at a flow rate of 1 mL/min in splitless mode, with an injection port temperature of 250 °C. Column oven temperature program settings were as follows: maintain at 40 °C for 3 min, then increase the temperature at a rate of 10 °C/min to 230 °C and maintain it for 5 min. We used the full scan acquisition mode, scanning range of *m/z* 30–550, ion source temperature 230 °C, transfer port temperature 280 °C, and quadrupole temperature 180 °C. We identified compounds based on the NIST14 spectral library, then we used standard samples to determine whether the retention times were consistent. We then calculated the relative mass fraction using the Area Normalisation Method [20].

### 2.4. Electroantennography (EAG)

Paraffin oil was used as the solvent to prepare solutions for each standard compound at concentrations of 0.01, 0.1, 1, 10, and 100 μg/μL. When not in use, the solutions were stored at −20 °C. Liquid paraffin was used as a control. All chemicals were purchased from Changchun Landel Technology Co., Ltd., Changchun, China. In preliminary tests, the lowest (0.01 μg/μL) and highest (100 μg/μL) concentrations were found to mark the response thresholds of bumblebees to the volatiles. Using EAG, volatile substances with a concentration of 1 μg/μL were initially screened, and then dose–response experiments were conducted on volatile substances that produced a strong electrical response in bumblebees during the initial screening. Before testing, adult bumblebees were starved for 3 h.

We applied an appropriate amount of conductive adhesive (SpectraR360, PPG Industries, Pittsburgh, PA, USA) to the EAG probe head and used a scalpel (Feather Safety Razor Co., Osaka, Japan) to cut off the pair of bumblebee antennae along the base, removed the scape, and then removed the front end of the antennal flagellum. We connected the antennae to the electrode coated with conductive adhesive, adjusted the position to ensure that both ends of the antennae were in full contact with the electrode surface, and we made sure that the antennae were not bent when detached from the body. The electroantennography air pump sent two test air streams to blow on the detached antennae simultaneously. One stream was a continuous air stream that had been filtered and humidified before being blown out, and the other stream was an air stream carrying the sample stimulus odor.

During testing, we took 10 μL of paraffin oil with the sample to be tested, mixed them evenly, and added them to the center of a 4 cm long × 0.2 cm wide strip of filter paper that was cut in advance. We then placed it in a Pasteur tube (paraffin oil is used to balance the odor). Before testing, we used a piece of hard cardboard to stimulate the detached antennae to detect their activity level. If there were significant fluctuations, we allowed the test to proceed. We waited until a stable baseline appeared on the recorder and used the foot pedal to trigger the stimulus.

The stimulus duration time was ~0.3 s, with intervals of more than 30 s between stimuli to allow the antennae’s sensory function to fully recover and minimize error. The stimulation sequence alternated between paraffin oil and the sample. The sample-testing sequence progressed from low to high concentration. Each sample was tested 4 times, and the test antennae were replaced after each sample was tested once. The test antennae were cut as needed to maintain their activity. Since the EAG response of the insect was relatively stable in the morning, all experiments were conducted between 7:00 and 11:00 a.m. The EAG reaction test results are expressed as the relative EAG reaction value of worker bees to the sample (m represents the Mean), as shown in Equation (1):

EAG relative response value = (m(Sample stimulation) − m(Contrast stimulation))/(m(Contrast stimulation)) × 100%(1)

### 2.5. Y-Tube Olfactometer Choice-Behavioral Assays

From the EAG reaction test results, volatile compounds with a significant stimulating effect on bumblebees were selected for behavioral assays. All 60 worker bees tested were randomly drawn from 15 colonies (ca. 300 bees each). The Y-tube had a main arm length of 24 cm, side arms of 26 cm angled at 60°, with an inner diameter of 4 cm for both the main arm and side arms. The two arms were connected to the odor source bottle and the control bottle, respectively. Before the airflow (200 mL min⁻¹) entered the odor source bottle and the control bottle, it was first passed through an activated carbon filter and a distilled water bottle to purify the air and increase the air humidity. We placed the test bumblebees in the middle of the Y-tube, one bumblebee at a time, and observed the bumblebees’ preferences for different odors and concentrations. We replaced the sample and control paraffin oil for every 10 bumblebees. Each concentration of an odor was tested on 60 bumblebees. The evaluation criteria were as follows: each worker bee was observed for a minimum of 2 min, and the longest time spent by a bumblebee entering the insect odor source bottle or remaining within the 1/3 area of the odor source bottle was considered a selection of that substance. When changing different odor sources, we wiped the inner and outer walls of the Y-tube with ultrapure water, allowed it to air dry, and only continued the test after no odor residue remained. The test duration was the same as in the EAG test, both conducted between 7:00 and 11:00 a.m.

### 2.6. Data Analysis

All statistical analyses and graphical representations were performed using GraphPad Prism 9 (Company: GraphPad Software Inc., San Diego, CA, USA). For the analysis of EAG response results, we used the one-way analysis of variance (ANOVA) and multiple comparisons to analyze significant differences. Behavioral responses were analyzed using the chi-square test. Differences were considered significant with *p* < 0.05.

## 3. Results

### 3.1. Analysis of the Types and Contents of Volatiles

The analysis of the types and contents of volatiles from GC–MS was employed to identify compounds compared to those in the NIST14 spectral library. Quantitative analysis was performed using the normalized total ion current peak area. As shown in Table 1, volatile compounds released by blueberry flowers belong to six categories and comprise 32 components, including 10 alcohols, 5 aldehydes, 4 esters, 1 ketone, 3 aromatic compounds, and 9 olefins. Among these, linalool and styrene were the primary components, with relative amounts of 25.93% and 14.28%, respectively.

### 3.2. EAG Responses

**Figure 1:**
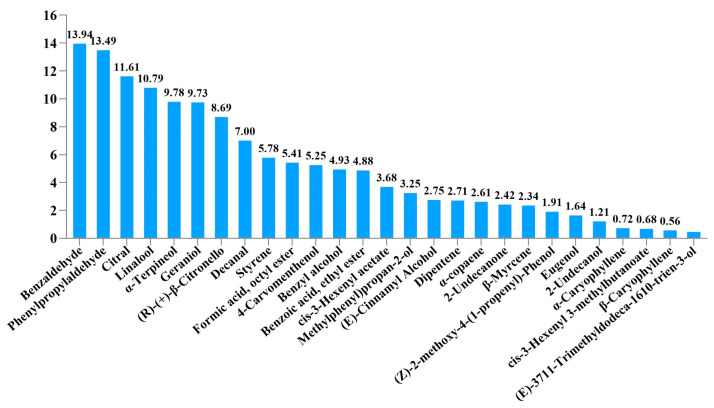
EAG relative response values of each volatile of *Vaccinium* spp. at a concentration of 1 μg/μL.

Among the 32 compounds detected in the airborne odor of blueberry flowers, only six elicited strong antennal electrical responses in bumblebees in the EAG experiment. Six compounds—benzaldehyde, phenylpropylaldehyde, citral, linalool, α-terpineol, and geraniol (Figure 1)—were identified as the key active constituents responsible for attracting pollinators. Among them, benzaldehyde caused a higher bumblebee antennal electrical response than geraniol.

**Figure 2:**
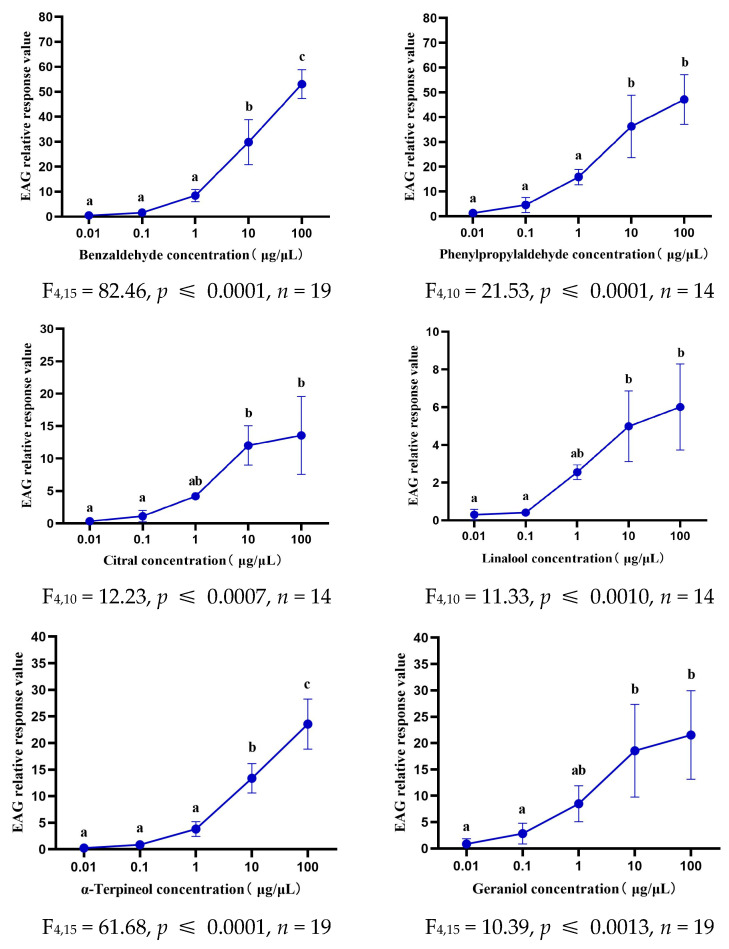
Dose–response of EAG active volatiles. Perform a one-way ANOVA analysis on the EAG response results. Significant differences are indicated by lowercase letters: the same letter indicates no difference, and different letters indicate significant differences *p* < 0.05.

For most test stimuli, the antennal response increased with increasing compound concentration. However, the thresholds for antennal electrical responses to different volatiles varied from bumblebee to bumblebee. Antennal responses to benzaldehyde and α-terpineol reached the maximum at 100 μg/μL, whereas sensitivity at 10 μg/μL was greater for phenylpropylaldehyde, citral, linalool, and geraniol (Figure 2).

### 3.3. Choice Behavioral Responses

**Figure 3:**
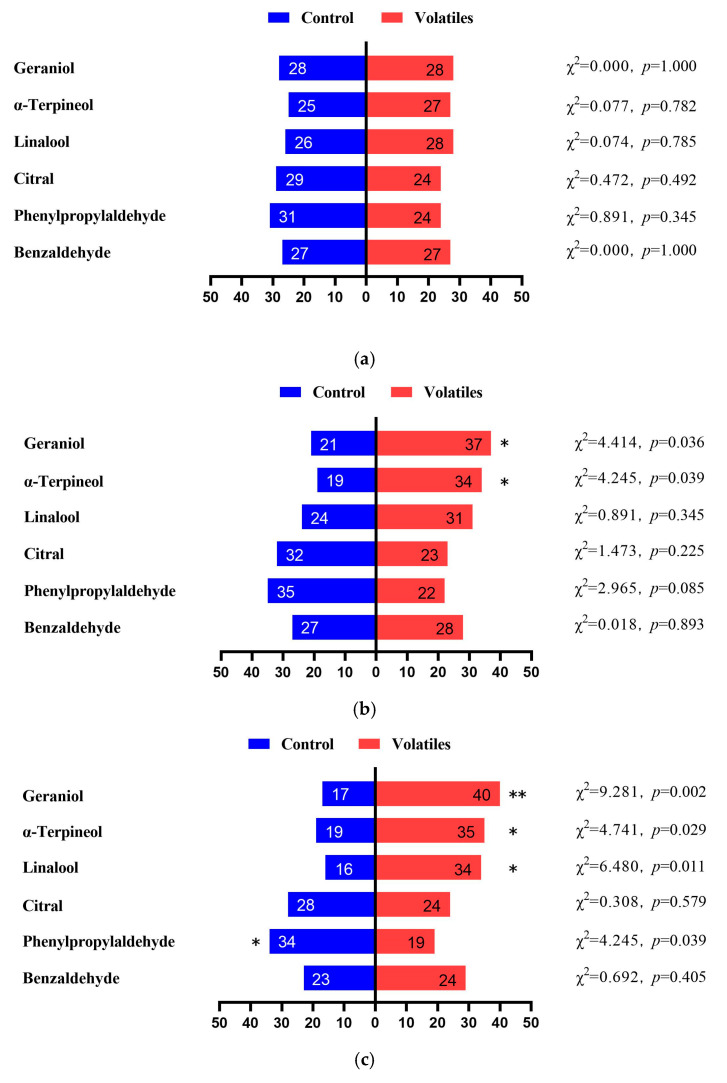

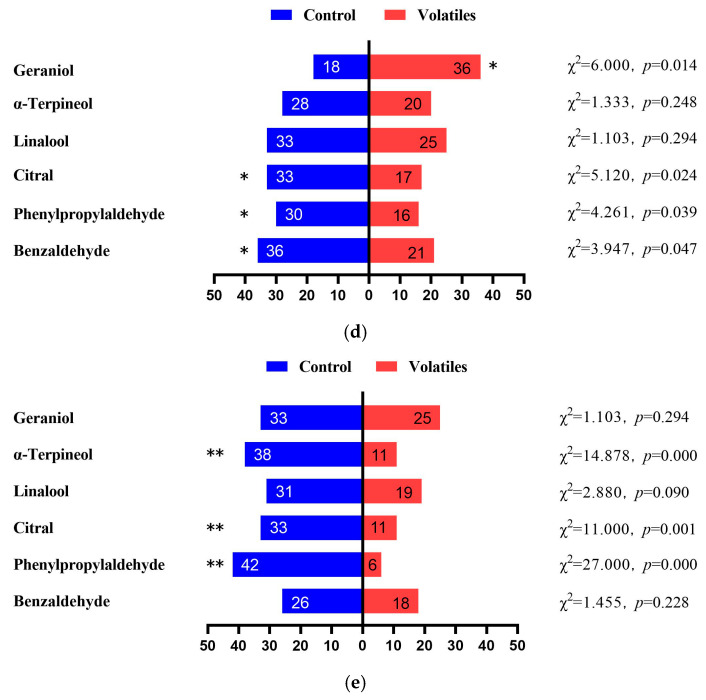
Behavioral responses of bumblebees to six volatiles. The corresponding volatile concentrations for (**a**–**e**) are 0.01 μg/μL, 0.1 μg/μL, 1 μg/μL, 10 μg/μL, and 100 μg/μL. The right side shows six volatile substances, while the left side shows the control (paraffin oil). Positive values on the right represent the attraction effect, and negative values on the left represent avoidance. The numbers in each bar chart represent the number of bumblebees that made the selection. In the chi-square test, * indicates a significant difference between volatile and control groups with *p* < 0.05, ** indicates a significant difference between the two with *p* < 0.01.

Six volatile compounds that elicited strong antennal electrical responses in bumblebees were used in olfactory and choice behavioral experiments. Bumblebees exhibited clear concentration-dependent responses to the six EAG-activating compounds tested. Overall, geraniol had a significant attractiveness to bumblebees at 0.1, 1, and 10 μg/μL (Figure 3b–d) and linalool only exhibited an attractive effect at 1 μg/μL (Figure 3c). However, α-terpineol exhibited attractiveness at 0.1 and 1 μg/μL, but repelled bumblebees at 100 μg/μL (Figure 3b,c,e). Phenylacetaldehyde repelled bumblebees at 1, 10, and 100 μg/μL (Figure 3c–e), and citral was repellent to bumblebees at 10 and 100 μg/μL (Figure 3d,e). However, benzaldehyde only caused bumblebees to exhibit clear repellency at 10 μg/μL (Figure 3d). In addition, the concentration of volatile repulsive compounds was generally higher than that of volatile attractive compounds (Figure 3).

## 4. Discussion

In nature, floral scent—composed of species-specific blends of volatile organic compounds (VOCs) released as secondary metabolites—acts together with visual cues to attract or orient pollinators [21,22,23]. These odors span fragrant and malodorous types, both of which promote pollination: sweet aromas enable bees and butterflies to locate nectar rewards, whereas fetid emissions attract flies or beetles [24,25,26,27]. Studies have demonstrated that bumblebees prefer brightly colored, aromatic flowers such as melon, mimosa, clematis, and tomato [28,29,30], underscoring the pivotal role of floral volatiles in mediating plant–pollinator interactions.

In our study, we employed GC–MS, EAG, and behavioral experiments to determine the electrophysiological and behavioral activity of blueberry floral volatiles on bumblebees. The active compounds identified are generally alcohols, aldehydes, esters, ketones, aromatic compounds, and olefins. Here, we detected six EAG-active compounds, including benzaldehyde, phenylpropyl aldehyde, citral, linalool, α-terpineol, and geraniol. Five volatile substances with significant effects were identified, i.e., linalool, cinnamyl alcohol, 1-pentene-3-ol, hexanal, and (E)-2-hexenal [31,32,33]. The reason for minimal chemical similarity may be due to differences in blueberry varieties and growing regions. A study by Rodriguez-Saona et al. showed that changes in blueberry flower volatiles depend on various factors, including pollination status, plant cultivation, and the time of day [34]. Another possibility is that discrepancies may be influenced by differences in cultivation, temperature, humidity, soil properties, analytical conditions employed, and resolution of the gas chromatography–mass spectrometry system [35].

In behavioral tests, bumblebees exhibited different behaviors in response to different volatiles. During evolution, bumblebees also developed a preference for floral chemical components related to their honey-gathering habits [36]. Olfactory signals are received by the antennae and then transmitted to the primary olfactory center, the antennal lobe [37,38]. The ovoid structure within the antennal lobe is the basic unit responsible for olfactory function. Different olfactory signals are processed through the spatiotemporal excitation patterns of specific ovoid structures [39] and then transmitted via nerves to the higher olfactory center, the mushroom body. Distinct characteristics of odors are processed separately by these nerve tracts [40,41]. In processing this olfactory signal, bumblebees are always interested in certain aromatic alcohols, but they show weaker responses or avoidance towards certain aromatic aldehydes, which is consistent with our research findings. Previous studies have shown that this may be due to these aldehydes not being commonly found in their environment, indicating that they are not food sources that lead to adaptive preferences for chemicals and highly specific olfactory receptors [7,42]. These physiological characteristics enable bumblebees to link ecological needs with behavioral habits, resulting in unique preferences for specific chemical components.

Insect behavior is associated with floral volatiles. Studies have shown that pollinators are attracted by floral color, size, olfactory cues, and nectar [43,44]. Many insects respond to either visual or olfactory stimuli alone [45,46,47], whereas bees rely on odor rather than visual cues for long-range orientation [45,48]. In the present study, we first collected and identified blueberry volatiles, and we then used EAG to rapidly screen compounds that elicited strong electrophysiological activity in *Bombus terrestris* and determined their optimal concentrations to narrow the scope of our behavioral assays. All our measurements were performed only using individual, not mixed, compounds. Given the large discrepancy between laboratory and field conditions, the active compounds should be blended at different ratios and systematically evaluated under greenhouse and open-field conditions for their effects on bumblebee visitation rate, pollination efficiency, and blueberry fruit set, so as to provide precise theoretical parameters and operational protocols for using *B. terrestris* in blueberry protected-culture pollination.

## 5. Conclusions

Floral fragrances play a crucial role in bumblebee pollination of plants. In this study, 6 classes totaling 32 volatile compounds were identified from blueberry flowers from Changbai Mountain. Here, we found that alcohols are the most abundant compounds identified. Different concentrations of these volatiles elicited clear electroantennographic (EAG) responses in Bombus terrestris. These bees showed strong EAG reactions to benzaldehyde, phenylpropyl aldehyde, citral, linalool, α-terpineol, and geraniol. Behavioral assays revealed that linalool, α-terpineol, and geraniol attracted the bumblebees, whereas phenylpropyl aldehyde repelled them, with higher concentrations of benzaldehyde and citral promoting bee avoidance.

## Figures and Tables

**Table 1:** Volatile compounds of *Vaccinium* spp.

CM	Compounds	CAS Number	Retain Time (s)	Relative Content (%)
**Alcohols**				
	Benzyl alcohol	100-51-6	9.028	0.92
	Linalool	78-70-6	10.419	25.93
	4-Carvomenthenol	562-74-3	11.727	2.36
	2-(4-Methylphenyl)propan-2-ol	1197-01-9	11.848	1.06
	α-Terpineol	98-55-5	11.948	3.92
	(R)-(+)-β-Citronello	1117-61-9	12.511	6.07
	Geraniol	106-24-1	12.923	5.78
	2-Undecanol	1653-30-1	13.569	3.32
	(E)-Cinnamyl Alcohol	4407-36-7	13.702	0.42
	(E)-3,7,11-Trimethyldodeca-1,6,10-trien-3-ol	40716-66-3	16.985	0.42
**Aldehydes**				
	Benzaldehyde	100-52-7	7.153	1.79
	Phenylacetaldehyde	122-78-1	9.194	1.64
	Phenylpropylaldehyde	104-53-0	11.461	0.64
	Decanal	112-31-2	12.123	0.43
	Citral	5392-40-5	12.702	0.58
**Esters**				
	cis-3-Hexenyl acetate	3681-71-8	8.424	1.20
	Formic acid, octyl ester	112-32-3	9.803	1.26
	Benzoic acid, ethyl ester	93-89-0	11.598	6.21
	cis-3-Hexenyl 3-methylbutanoate	35154-45-1	12.59	1.80
**Ketones**				
	2-Undecanone	112-12-9	13.452	2.06
**Aromatic compounds**				
	m-Cymene	535-77-3	8.769	2.92
	Eugenol	97-53-0	14.406	2.89
	methoxy-4-(1-propenyl)-Phenol	5912-86-7	15.631	0.46
**Olefins**				
	Styrene	100-42-5	4.812	14.28
	β-Myrcene	123-35-3	8.024	2.09
	Dipentene	138-86-3	8.857	3.87
	α-Cubebene	17699-14-8	14.319	0.44
	α-copaene	3856-25-5	14.706	0.99
	(-)-β-Bourbonene	5208-59-3	14.844	0.75
	β-Caryophyllene	87-44-5	15.319	1.61
	α-Caryophyllene	6753-98-6	15.761	0.81
	γ-Muurolene	30021-74-0	16.015	0.29

## Data Availability

All data are available in this paper.
